# Comparison of Certain Intrarectal versus Intramuscular Pharmacodynamic Effects of Ketamine, Dexmedetomidine and Midazolam in Cats

**DOI:** 10.3390/vetsci9100520

**Published:** 2022-09-23

**Authors:** Andrea Paolini, Massimo Vignoli, Giulia Guerri, Ilaria Falerno, Roberto Tamburro, Francesco Simeoni, Francesca Del Signore, Andrea De Bonis, Francesco Collivignarelli, Maria Cristina Salvo, Ilaria Cerasoli

**Affiliations:** 1 Faculty of Veterinary Medicine, University of Teramo, Località Piano D’Accio, 64100 Teramo, Italy; 2 Clinica Veterinaria Borghesiana, 00132 Rome, Italy

**Keywords:** cats, intrarectal route, intramuscular route, dexmedetomidine, ketamine, midazolam, sedative effects

## Abstract

**Simple Summary:**

The chemical immobilization of cats is widely required in veterinary clinical practice; sedative drugs are administered intramuscularly and routinely, but this is painful and uncomfortable. The intrarectal route is commonly used in humans for the sedation of uncooperative patients and is very safe, but it is not investigated in cats. In the present study, twenty owned cats were included, ten underwent intramuscular sedation, and the other ten intrarectal sedation. Cardiorespiratory values, pulse oximetry, body temperature, sedation score, and the feasibility of venous catheter placement were compared between the two groups at pre-established time points. Cats that received the intrarectal administration showed a shorter and superficial state of sedation than intramuscular ones, but in the intrarectal group, the maintenance of SpO₂ values was >95% during the experimental period and the recovery of the quadrupedal station was faster. According to these results, the intrarectal route appears to have a high efficacious option for performing minimally invasive clinical and diagnostic procedures in cats.

**Abstract:**

The aim of this clinical trial was to evaluate the impacts of administration via the intrarectal route (IR) in cats on their heart and respiratory rates, blood pressure, body temperature, and sedation quality compared to the intramuscular route (IM). The intramuscular group (IMG) received 0.003 mg kg^−1^ dexmedetomidine, 2 mg kg^−1^ ketamine, and 0.2 mg kg^−1^ midazolam while the intrarectal group (IRG) protocol was 0.003 mg kg^−1^ dexmedetomidine, 4 mg kg^−1^ ketamine, and 0.4 mg kg^−1^ midazolam. Cardiorespiratory values, temperature, and sedation score were measured 2 min after administration and then every 5 min up to the 40th minute. Cats belonging to IRG reacted less strongly to the drug, as opposed to those receiving intramuscular administration (2/10 in IRG vs. 8/10 in IMG). Average time between drug administration and standing position was 44.9 ± 5.79 in IRG and 57 ± 9.88 min in IMG. In IRG, maintenance of SpO₂ values is >95% at each time point. Median and range peak of sedation {7 (5)} in IMG occurs at 20th, 25th, and 30th minutes post drug administration while was lower in IRG. Cardiorespiratory values were slightly lower in IMG than in IRG, but always constant in both treatments. Temperature did not differ between groups. At this dosage, although sedation score was higher in IMG, intrarectal route could be efficacious for performing minimally invasive clinical and diagnostic procedures in cats.

## 1. Introduction

Sedation is often required in cats for investigations or diagnostic procedures, which can be uncomfortable and painful, to improve cooperation, animal comfort, and personal safety [1,2]. Chemical immobilization can be required for blood sampling, X-rays, biopsy, or other procedures [1]. The intrarectal route (IR) is widely used in human medicine for the sedation of uncooperative patients, such as children, and has wide safety margins [3]. Common routes of sedation in cats are intramuscular (IM), intravenous (IV), and oral (OS). In humans, IM injection is the most painful and uncomfortable route of drug administration [4]. The extrapolation of these findings to cats suggests that alternatives to IM administration could be useful in veterinary practice; particularly when multiple injections are necessary, the IR route could be an alternative. Per rectum, drugs can partially avoid hepatic metabolism following systemic absorption, which reduces the first-pass hepatic effect observed with OS administration [5]. Thus, IR administration can provide significant local and systemic levels for various drugs, despite the relatively small surface area of the rectal mucosa [6,7]. In human medicine, IR administration is used for the treatment of epileptic seizures [3,6], during pediatric sedation, for burn wounds [8], for vomiting, and for dysphagia patients [9]. In veterinary medicine, IR administration is extensively investigated for the control of epileptic seizures in dogs, but less so in cats [10,11]. Unfortunately, there is still insufficient knowledge in the veterinary literature on the bioavailability of opioids, dexmedetomidine, and ketamine after IR administration [12,13]. Compared to other routes, it could have some advantages: faster and more predictable sedation action than OS administration, less physical trauma, and it is less painful than the IM and IV routes [14]. Thus, stress is reduced for cats, owners, and veterinary surgeons. Alternatives to IM injection could be useful in veterinary practice, especially when considering long and repeated therapies throughout the day in hospitalized cats [15]. In this study, we compared a different solution of dexmedetomidine, ketamine, and midazolam for chemical immobilization by the IM and IR routes. We hypothesize that IR administration could elicit a sedation, similar to the IM one, with less of a reaction upon administration. At the proposed dosages, we assume a minimal cardiovascular and respiratory response in both groups.

## 2. Materials and Methods

This study was approved by Committee on Animal Research and Ethics of the Universities of Chieti–Pescara, Teramo, L’Aquila and of the Experimental Zooprophylactic Institute of Abruzzo-Molise (CEISA) Prot. N°8 3 March 2020. A group of twenty owned cats were brought to the Teaching Veterinary Hospital of the Faculty of Veterinary Medicine of Teramo that required chemical immobilization for small procedures. Owners were notified and asked to sign a written owner consent form. Cats were blindly and randomly assigned to the IR group (IRG) or IM group (IMG) through a randomization website (www.randomizer.org, accessed on 5 May 2021). Drug administration was performed by two of the same veterinarians that were not involved in data collection and were blind to drug solution. Clinical history and HR, pulse quality, *f*R, and type of breathing were clinically evaluated and recorded. Systolic, mean, and diastolic (SAP, MAP, and DAP) non-invasive arterial blood pressure (NIBP) were registered by a high definition oscillometry detector (HDO; S+B medVet GmbH, Babenhausen Germany) and rectal temperature was collected by digital thermometer (MT4233; Sejoy Electronics & Instruments Co., Hangzhou, China). According to the American Society Anesthesiologists (ASA), inclusion criteria were cats classified as ASA I or II [16]. Cats with rectal and perineal pathologies classified as ASA II were excluded in this study. Animals for whom IR or IM administration was failed were not included. Fasting for sedation was 8 h, but water was allowed until the procedure began. The IMG received dexmedetomidine (Dextroquillan; Fatro, Bologna, Italy) (0.003 mg kg^−1^), ketamine (2 mg kg^−1^) (Ketavet; MSD Animal Health, Kenilworth, NJ, USA) and midazolam (0.2 mg kg^−1^) (Midazolam; Pharma Hameln, Hamelin, Germany); the IRG received dexmedetomidine (0.003 mg kg^−1^) (Dextroquillan; Fatro, Bologna, Italy), ketamine (4 mg kg^−1^) (Ketavet; MSD Animal Health, Kenilworth, NJ, USA) and midazolam (0.4 mg kg^−1^) (Midazolam; Pharma Hameln GmbH, Hamelin, Germany). IR administration was performed by insulin syringe (Micro-fine 1 mL; BD, New York, NY, USA) 0.3 mL inside the rectum, without needle, while IM injection was carried out with the same model of insulin syringe on the longissimus dorsi muscle. No enema or rectal emptying was performed before drug administration. Reaction to administration was assessed by a different operator than the one who administered the drugs. Subjects who showed at least two of the following behaviours during administration were considered positive cases: vocalizations, attempts to retreat, aggression, or inability to restrain. Purring was excluded as a factor to be considered. After 2 min post administration, and then every 5 up to 40 min, the following variables were evaluated: HR, *f*R, SpO2, SAP, MAP, and DAP. HR was recorded from the femoral artery; the NIBP cuff was positioned at the base of the tail. In case of bradyarrhythmias, atipamezole IM (Revertor; Cp Pharma Mbh, Burgdorf, Germany) was administered at 2.5× previous dexmedetomidine dose and flumazenil (Anexate; Help a Pharm GmbH, Hamelin, Germany) (0.02 mg kg^−1^) IV was administered as reversal dexmedetomidine and midazolam, respectively. A*f*R was detected by clinical observation of chest movements. A pulse oximeter was applied to the tongue and measured SpO2 with a multiparametric monitor (M3046-M2, Philips, Amsterdam, Netherlands) when possible. Oxygen was administered at 150 mL kg^−1^ min^−1^ through a Mapleson C system (Mapleson C rebreathing system, Intersurgical Ltd., Berkshire, UK) if SpO2 was lower than 95%. In case of apnea, the trachea was intubated with a PCV cuffed endotracheal tube (Rusch; The Sheridan, Morrisville, NC, USA). Rectal temperature was measured at 15, 30, and 40 min post drug administration and at the standing position. At the fifteenth minute post administration, a 22-gauge catheter was inserted in the cephalic vein (Jelco; Smiths Medical, Minneapolis, MN, USA) and Lactated Ringer’s solution (Baxter Healthcare Corp, Deerfield, IL, USA) was then administered at rate of 3 mL kg^−1^ h^−1^. It was also recorded whether the placement of the intravenous catheter on the cephalic vein was possible or not. Recovery time was recorded from the fortieth minute to the resumption of the standing position. Sedation score was assessed after 2, 5, 10, 15, 20, 25, 30, 35, and 40 min post drug administration by sedative scale [15], where zero was no sedation and eight was no response to acoustic and tactile stimuli; so higher scores indicated greater sedation (Table 1).

### Statistical Analyses

Data analyses were performed using the appropriate statistical software R and the following packages: ggpubr and psych. All data were tested for normality using the Shapiro–Wilk test. The demographic data of age, weight, and time elapsed from starting sedation to achievement of standing position were presented as media and standard deviation (SD). An exact Wilcoxon–Mann–Whitney test was performed to verify there were no significant differences between groups at T0. To compare HR, *f*R, SpO2, SAP, MAP, DAP, T, and sedation scores over time, IRG and IMG were analyzed using a two-way repeated measures ANOVA for normally distributed data and the aligned rank transform test for non-normally distributed data. Sidak’s multiple comparison test and the Mann–Whitney u test (Holm’s method) were used post hoc among different times in each group for normally and non-normally distributed data, respectively. Data are expressed as mean and SD when normally distributed or median and range if non-normally distributed. The information of cats’ reactions to drug administration and successful vein catheterization is presented as a percentage and was analyzed with Fisher’s exact test. Significance level was set a *p* value ≤ 0.05

## 3. Results

Mean age is 5.55 years (SD ± 3.82) and mean body weight is 4.13 kg (SD ± 1.14) in the IMG, 4.60 years, and 4.54 kg (SD ± 1.12) in the IRG. Baseline vital values show no significant difference between cats (Table 2).

Among clinical variables monitored during sedation, some showed significant differences between groups, this is shown in the table (Table 3).

The sedation level is presented as median, and range is significantly higher in the IMG than the IRG at minute 10 (*p* = 0.02), 15 (*p* = 0.03), 20 (*p* = 0.02), 25 (*p* = 0.01), 30 (*p* = 0.02), 35 (*p* = 0.02), and at 40 min (*p* = 0.03). In the IMG maximum, sedation scores are achieved: 7.0 (5) at minute 20, 7.0 (5) at 25, and 7.0 (6) at 30. This is significantly higher than the value obtained in the IRG, in which the maximum peak of the sedation score reached is 3.5 (6) at the minute 15, post administration (shown in Table 4).

**Figure 1:**
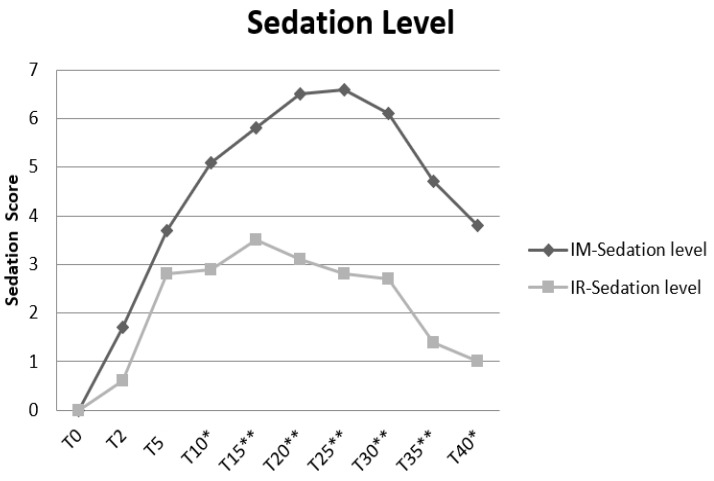
Trend of sedation levels in intrarectal (IRG) and intramuscular group (IMG). The table shows the trend of sedation curve during the time of procedure. Sedation score is along the ordinate, while time is along the abscissa. The graph shows increasing sedation in both groups over the time to the fifteenth minute post administration. Asterisk shows significance of value. Significative level is set to 0.05. *, ** significant results in table.

As far as rectal temperature is concerned, there were no significant changes. The temperature records at 15, 30, and 40 min are never dropped below 36.5 Celsius degrees (C°) in both groups. For each cat, the temperature at the time of standing position was comparable to the pre-administration measurement, but this is not statistically significative (*p* value = 0.081). The average time from the beginning of the procedure to the recovery of the standing position is presented as mean and SD (57 ± 9.88 min in IMG and 44.9 ± 5.79 in IRG). All IMG cats lost the four-footed station, unlike IRG, where two cats never lost. For IM, eight out ten of cats reacted to administration, unlike animals who received IR that reacted only in two cases (*p* = 0.02). In the IMG, increasing sedation levels are recorded from the fifteenth (median level score 6) to the thirtieth minutes (median level score 7). In the IRG, the sedation level decreased at the fifteenth minute, from a 3.5 median level score to 2 at the thirtieth minute. The insertion of the venous catheter at minute 15, post administration, was performed in 100% of cases in the IMG and in 60% of cases in the IRG (*p* = 0.08). Flow by oxygen is required in five of ten animals of IMG; in contrast, none of the cats in the IRG need it (Figure 1).

## 4. Discussion

The feline species are easily stressed animals. Different routes of administration could elicit different pharmacokinetic and pharmacodynamic responses of the same drug mixture. A decreased uptake of drugs is already known for benzodiazepine in dogs for IR; as suggested by [11,17], similar clinical effects occur with a range dose of 0.5–2 mg kg^−1^ in IR and 0.2–0.3 mg kg^−1^ in IV administration of diazepam. This consideration would seem valid for midazolam: IV and IR at the same posology present lower bioavailability in the second case [18]. It is shown to be a more hydrophilic molecule than other benzodiazepines and if its ambient pH does not increase towards a more neutral one, its transmucosal uptake in dogs is lower [18]. Different studies also show a different drug deposition site in the rectum [18,19]. The advantage of IR is that it partially skips the first-pass effect of hepatic metabolism. However, the venous drainage of the canine and feline rectum occurs in two main areas: the caudal and middle rectal vein through the caudal vena cava with the bypass of hepatic metabolism and the cranial rectal veins, which instead drain through the portal vein into the liver [20]. Therefore, for the same dosage, depending on where the drug is released into the rectum, there may be a different amount of absorption, so a higher dose in the IR route is required to be efficacious.

A pharmacokinetics study of ketamine is described previously in the literature. It suggests that higher doses for IR could be required in cats. In our study, the clinical effects of ketamine were observed at lower dosages (4 mg kg^−1^) than those reported in the bibliography (25 mg kg^−1^) [12]. IR midazolam and dexmedetomidine in cats, which to compare the dosage proposed in this study, is not present in bibliography. According to the literature for ketamine and buprenorphine in pharmacokinetics and pharmacodynamics studies, we decided to choose a higher dosage for IRG than IMG. However, due to the lack of studies on the pharmacokinetic and pharmacodynamic of dexmedetomidine and midazolam in cats for this route, authors proposed an “alfa dose” to investigate. As evidenced by the results, this drug mixture led to a brief but efficacious sedation with minimal systemic effects. In the present study, the cardiovascular effects of dexmedetomidine, which consist of an initial increase in blood pressure accompanied by bradycardia and a decrease in blood pressure that occurs approximately 20 min after administration, were more evident in the IMG [21,22,23,24]. On the other hand, for cats treated intrarectally, cardiovascular values remained almost constant, different from those of the IMG, particularly at minute 25, coinciding with the hypotensive peak observed in the latter group. This could be related to the different pharmacokinetics of dexmedetomidine when administered intrarectally. At a dosage of 0.003 mg kg^−1^ par rectum, typical alfa 2 agonist cardiovascular effects were not observed. The hepatic metabolism of administered drugs involves oxidative processes that intervene in a linear manner with hepatic perfusion. IM and IV alfa 2 agonist administration cause a decrease in cardiac output in a dose-dependent manner [25]. The IR dexmedetomidine absorption might be different from usual routes, such as IM and IV, but this is only a speculation, as it is not supported by a pharmacokinetic study. Modifications to HR and blood pressure, as well as sedation scores, were lower in the IRG compared to the control group. This could also influence the metabolization of the drugs in the liver and explain the increase and prolongation in the intensity of the clinical effects in the IMG compared to the experimental group, where the cardiovascular effects of dexmedetomidine are imperceptible. Instead, there is an increase in HR and SAP, probably linked to the administration of ketamine, which causes the inhibition of the re-uptake of adrenaline and noradrenaline, thus causing an increase in their plasma concentration and receptor availability. This is manifested in a positive inotropic, chronotropic, and bathmotropic effect at the cardiac level, as well as in an increase in systemic arterial pressure. Consequently, there will be a decrease in the volumes of distribution and an increased metabolization of drugs [12,26]. As far as sedation is concerned, the same consideration could be applied because of the lack of alfa 2 agonist absorption from the rectum.

In human medicine, there are several studies on the pharmacokinetics of diazepam [27], ketamine [28,29], and midazolam [30] administered IR, while there are none for dexmedetomidine. From the results obtained in this first study, it is possible to hypothesize a lack of or poor absorption of dexmedetomidine by IR route. As for intraoral administration, the physicochemical characteristics of the molecule could result in reduced absorption through the rectal mucosa [15]. An increase in doses could lead to clinical effects comparable to those of the IM route, as demonstrated by the transmucosal route in healthy cats in combination with buprenorphine [31] and encouraging effects of IR route in cats for analgesia [13].

Cats represent a numerically large population of pets and are easily stressed animals [1]. It is documented that owners have a significant perception of discomfort when bringing their animals to the veterinarian [2]. Vital signs and laboratory test results can reflect the effects of discomfort and be difficult to interpret. Additionally, any vet visits associated with anxiety and distress can lead cats to expect future visits to be similar [32].

For these reasons, different strategies were explored to reduce animal anxiety and increase compliance. Feline-friendly handling techniques, behavioral conditioning, using an appropriate examination room and anxiolytic drugs, such as trazodone, gabapentin, and dexmedetomidine, are among the most commonly used strategies for these animals, but are not always effective [32,33]. Sedation is often necessary in cats where levels of stress and pain result in fear, aggression, and or inability to contain and manage them [33]. IM injections are generally stressful and painful; a pain stimulus, aside from the sting itself, can also originate from low-pH mixtures. Ketamine is considered because it is commercially available in acidic pH mixtures (pH 3.5–5.5) and is a registered and widely used IM. In addition, repeated SC or IM injections increase the risk of incidence of malignant neoformations, such as fibrosarcoma [34,35]. An easy and atraumatic route of administration, such as IR would ensure minimal pain-free stimulation and stress on the part of the cat. An atraumatic administration would also ensure the minimal activation of the sympathetic fight–flight system. During sedation, this would decrease the interaction between endogenous neurotransmitters and any competitive drugs for the same receptor site [36] (such as alpha 2 agonists), ensuring better and faster sedation with a lower dosage of drug. For this reason, no enema was performed. In fact, the enema is a possible source of stress, as well as potential irritation, or the presence of residual emollients with the consequent modification of the environmental pH of the rectum and interaction in the reuptake of drugs administered. At the same time, the authors considered that the presence of stool in the rectum could interfere with the administration of the drugs.

The proposed mixture of drugs is used to ensure short-term sedation for procedures that required chemical immobilization with mild to moderate analgesic coverage. It should also be considered that the procedures performed on cats were essentially of three types: jugular samples, X-ray, and ultrasound studies with cytological sampling. Although minimal, the sensory and algic stimuli of the animals on the listed procedures are different; this can potentially cause a certain variability in cats’ responses.

The study design involved the placement of the venous catheter at the fifteenth minute, regardless of the sedation status of the animals (median of 3.5 in the IRG and 6 for the IMG). The state of sedation decreased markedly only in the IRG; in particular, six cats reacted to the attempt to place the catheter, four of which did not allow us to place it. In this way, sedation in the IRG was stopped prematurely. By waiting for a higher state of sedation, we probably would have a higher catheter insertion rate.

Finally, the person who evaluated the reaction to drug administration was aware of the administration route. In any case, cats’ reactions, meeting two parameters out of the four evaluated, tended to reduce the subjectivity of the operator. In this study, no drugs had to be antagonized. Flow-bay oxygen administration was required in five cats of the IMG. The cardiorespiratory stability found in the IRG may suggest that it could potentially be a valid approach to fearful or aggressive animals with cardiac, renal, hepatic insufficiencies, or generally with severely compromised systems (ASA status ≥ III). In addition, the onset of the IR effect appears to be faster than IM; this could be important to ensure a venous access can be gained as soon as possible in critically ill cats. In this regard, pharmacokinetic and pharmacodynamic studies are necessary to determine the useful dosages to maximize the sedative and analgesic effects exerted by the IR mix.

## 5. Conclusions

This is the first study to show that the sedation status of cats treated with IM administration is higher than that of IR-treated cats. This study also suggests that IR sedation could be an effective practice in cats, and less stressful than IM injection, and it is suitable for performing minimally invasive clinical diagnostic procedures, so further studies are needed to evaluate the pharmacokinetics and pharmacodynamics of dexmedetomidine, ketamine, and midazolam in cats.

## Figures and Tables

**Table 1:** Criteria for evaluating the level of sedation in cats: assessment score and definition (Santos et al., 2010). Evaluation criteria are: posture, response to the sound of clippers, response to clipping, and response to restraint, where 0 is no sedation and 8 is no response to acoustic and tactile stimuli. Lower score is bad sedation and higher scores indicate greater sedation.

Assessement	Score and Definition
Posture	0: Standing position, walking.
	1: In sternal or lateral position but stands when stimulated
	2: Remains in sternal recumbency; resists lateral recumbency
	3: Remains in lateral recumbency but might lift head
	4: Remains in lateral recumbency even when stimulated; flat out
Response to clipper sounds	0: Reacts strongly when clippers are turned on
	1: Reacts mildly when clippers are turned on
	2: No response when clippers are turned on
Response to clipping	0: Reacts strongly when hair is clipped
	1: Reacts mildly when hair is clipped
	2: No response when hair is clipped
Response to restraint	0: Alert, readily reacts to sounds and resists restraint (looks, lifts head)
	1: Alert but minimally responds to sounds and restraint (appears sedated)
	2: No reaction or movement in response to sounds or restraint

**Table 2:** Vital signs at general examination (pre-drug administration, T0 time point) in the intramuscular group (IMG) and rectal group (IRG). Respiratory rate (*f*R), heart rate (HR), sistolic (MAP), mean (MAP), and diastolic (DAP) pressure are presented by mean and standard deviation in IMG and IRG and relative *p* value (any statistically difference).

Vital Sign	IMG	IRG	*p* Value ≤ 0.05
*f*R (arm)	43.6 ± 3.26	47.2 ± 16.31	0.830
HR (bpm)	185 ± 24.8	176 ± 24.7	0.437
SAP (mmHg)	157 ± 33.89	159 ± 31.1	0.805
MAP (mmHg)	99.3 ± 22.9	101.6 ± 22.8	0.608
DAP (mmHg)	76.1 ± 21.60	74.8 ± 9.81	0.684

**Table 3:** Table shows the trend of vital variables post drug administration in the intramuscular group (IMG) and intrarectal group (IRG) during the experimental study. Values were collected from 2 min post administration to up fortieth minute. On the left, the time is expressed in minutes (t) of each registration (2, 5, 10, 15, 20, 25, 30, 35, and 40 after drug administration).

	Heart Rate			*f*R			SAP			MAP			DAP		
*t*	IM	IR	*p*	IM	IR	*p*	IM	IR	*p*	IM	IR	*p*	IM	IR	*p*
2	180 ± 30.9	173 ± 31.2	0.649	46.0 ± 20.2	51.6± 23.1	0.732	167 ± 27.9	150 ± 18.9	0.297	113 ± 23.6	98.0 ± 23.3	0.27	91.8 ± 24.6	74.2 ± 31.2	0.747
5	170 ± 24.4	160 ± 31.1	0.413	42.0 ± 21.6	54.2± 23.4	0.210	165 ± 30.3	152 ± 30.3	0.417	111 ± 22.2	106 ± 25.3	0.57	89.8 ± 22.2	79.1 ± 27.5	0.248
10	147 ± 25.6	160 ± 26.6	0.276	34.0 ± 24.2	46.4± 16.7	0.027 *	151 ± 23.5	153 ± 36.0	0.364	93.3 ± 20.5	99.7 ± 30.4	0.589	71.5 ± 19.3	71.4 ± 30.7	0.929
15	136 ± 24.2	154 ± 25.9	0.122	27.2 ± 12.3	43.4± 20.5	0.062	149 ± 31.1	151 ± 38.4	0.787	97.1 ± 29.3	97.9 ± 32.7	0.82	74.6 ± 26.3	70.6 ± 26.9	0.790
20	132 ± 21.0	165 ± 25.2	0.010 *	22.0 ± 8.69	42.8± 18.9	0.003 *	139 ± 23.9	155 ± 21.1	0.143	90.2 ± 26.5	99.2 ± 23.1	0.205	65.4 ± 19.4	75.0 ± 24.3	0.494
25	132 ± 22.5	174 ± 45.8	0.014 *	23.6 ± 9.51	46.4 ± 19.5	0.004 *	131 ± 19.4	158 ± 25.1	0.020 *	78.4 ± 16.9	110 ± 22.3	0.009 *	56.6 ± 16.0	92.4 ± 21.2	0.01 *
30	130 ± 26.1	166 ± 43.4	0.041 *	23.8 ± 9.64	47.6 ± 24.0	0.012 *	140 ± 22.7	151 ± 20.9	0.322	78.8 ± 17.5	93.3 ± 24.4	0.17	58.9 ± 12.4	80.7 ± 37.3	0.143
35	134 ± 27.1	172 ± 50.4	0.054	27.6 ± 13.3	48.8 ± 22.0	0.009 *	145 ± 22.5	158 ± 28.6	0.451	81.6 ± 13.3	105 ± 33.4	0.076	59.7 ± 13.3	82.7 ± 35.7	0.143
40	142 ± 34.8	176 ± 47.4	0.084	31.2 ± 14.8	47.6 ± 24.8	0.073	142 ± 20.9	156 ± 28.9	0.207	83.8 ± 14.7	98.7 ± 27.4	0.176	65.6 ± 14.9	81.6 ± 23.5	0.223

From left to right: vital signs and, respectively, *p* value (*p*) at each time of control IMG and IRG: heart rate (HR), respiratory rate (*f*R), systolic, mean and diastolic pressure (SAP, MAP, and DAP). Statistically significant values with relative *p* value (significant level < 0.05) are marked with an asterisk (*).

**Table 4:** Median and range of sedation score in comparison of group receiving intrarectal (IRG) and intramuscular (IMG) administration and relative *p* value. On the left: time post administration expressed in minutes. Range is reported between round brackets.

t	Median and Range Sedation Score		
	IMG	IRG	*p*
0	0 (0)	0 (0)	0.900
2	1.00 (7)	0.50 (2)	0.3600
5	3.00 (6)	2.00 (8)	0.36
10	4.50 (5)	2.50 (6)	0.02 *
15	6.00 (5)	3.50 (6)	0.03 *
20	7.00 (5)	3.00 (4)	0.02 *
25	7.00 (5)	3.00 (5)	0.01 *
30	7.00 (6)	2.00 (5)	0.02 *
35	5.00 (6)	1.00 (5)	0.02 *
40	3.50 (5)	0.50 (4)	0.03 *

*: Statistically significant results are marked with an asterisk.

## Data Availability

The data generated in this study is already added in the tables of this article. If you need any further information, please feel free to contact authors.
